# Uric Acid-to-HDL Cholesterol Ratio Is Associated with Prediabetes and an Adverse Metabolic Profile: Results from the Genetics of Atherosclerotic Disease (GEA) Study

**DOI:** 10.3390/diagnostics16132038

**Published:** 2026-06-30

**Authors:** Guillermo C. Cardoso-Saldaña, Rosalinda Posadas-Sánchez, María Fernanda Suárez-Velázquez, Jesús Ernesto Martínez-Luna, Juan Reyes-Barrera, Gilberto Vargas-Alarcón, Neftali Eduardo Antonio-Villa, Giovanny Fuentevilla-Álvarez

**Affiliations:** 1 Department of Endocrinology, Instituto Nacional de Cardiología Ignacio Chávez, Juan Badiano No. 1, Colonia Sección XVI, Tlalpan, Mexico City C.P. 14080, Mexico; gccardosos@yahoo.com (G.C.C.-S.); rossy_posadas_s@yahoo.it (R.P.-S.); reyesbarrera_juan@hotmail.com (J.R.-B.); 2 Facultad de Medicina, Universidad Nacional Autónoma de México, Circuito Interior S/N, Ciudad Universitaria (CU), Alcaldía Coyoacán, Ciudad de México C.P. 04510, Mexico; fersuvel@gmail.com (M.F.S.-V.); ernestoluna1911@gmail.com (J.E.M.-L.); 3 Department of Molecular Biology and Research Direction, Instituto Nacional de Cardiología Ignacio Chávez, Juan Badiano No. 1, Colonia Sección XVI, Tlalpan, Mexico City C.P. 14080, Mexico; gvargas63@yahoo.com

**Keywords:** uric acid-to-HDL cholesterol ratio, prediabetes, insulin resistance, cardiometabolic risk, adiposity, Mexican-Mestizo population

## Abstract

**Aims:** The uric acid-to-high-density lipoprotein cholesterol ratio (UHR) has emerged as a potential marker of cardiometabolic dysfunction. However, evidence regarding its association with prediabetes remains limited in Hispanic populations. We evaluated the association between UHR and prediabetes in Mexican-Mestizo adults from the Genetics of Atherosclerotic Disease (GEA) study. **Methods:** This retrospective cross-sectional analysis included 1395 adults without diabetes or coronary artery disease. Prediabetes was defined as fasting glucose levels between 100 and 125 mg/dL. Participants were stratified into sex-specific UHR tertiles. Logistic regression models adjusted for demographic, lifestyle, and cardiometabolic variables were used to evaluate the association between UHR and prediabetes. **Results:** Mean age was 53 ± 6 years, 51% were women, and 17% had prediabetes. Individuals in the highest UHR tertile exhibited higher adiposity indices, triglycerides, Apo-B, hsCRP, HOMA-IR, and lower HDL-C and adiponectin levels. Prediabetes prevalence increased progressively across UHR tertiles (11%, 15%, and 24%; *p* < 0.001). After multivariable adjustment, each 0.1-unit increase in UHR was associated with prediabetes (OR 1.65, 95% CI 1.26–2.17). Compared with the lowest tertile, individuals in the highest UHR tertile had more than two-fold higher odds of prediabetes (OR 2.03, 95% CI 1.36–3.05, *p* < 0.001). A significant interaction between UHR and sex was observed, with a stronger association in women than in men (OR 3.39, 95% CI 2.1–5.5 vs. 1.19, 95% CI 0.84–1.67). **Conclusions:** Higher UHR was independently associated with prediabetes and an adverse cardiometabolic profile in Mexican-Mestizo adults. UHR may serve as a simple complementary marker of glucose metabolic disturbances.

## 1. Introduction

The global prevalence of prediabetes has increased substantially over the last few decades and is expected to continue rising in the coming years. According to the International Diabetes Federation, approximately 5.8% of the world population had prediabetes in 2021, with projections reaching 6.5% by 2045 [1]. However, important ethnic and regional differences exist. Hispanic and Latino populations consistently show higher prevalence and incidence rates of prediabetes compared with non-Hispanic White populations [2]. In Mexico, data from the 2022 ENSANUT survey estimated that nearly 22% of adults meet criteria for prediabetes [3].

The clinical relevance of prediabetes extends beyond its role as a precursor stage of type 2 diabetes. Previous studies have shown that individuals with prediabetes have increased risk of cardiovascular disease, renal dysfunction, and all-cause mortality, even before the development of overt diabetes [4]. These adverse outcomes are unlikely to be explained solely by mild hyperglycemia and are probably reflective of a broader cardiometabolic disturbance involving insulin resistance, chronic low-grade inflammation, adipose tissue dysfunction, and alterations in lipoprotein metabolism [5].

Emerging evidence suggests that chronic low-grade inflammation originating from adipose tissue plays a central role in the early stages of glucose dysregulation. Pro-inflammatory cytokines, such as tumor necrosis factor-alpha (TNF-α), have been associated with impaired insulin signaling, adipose tissue dysfunction, and alterations in lipid metabolism, whereas adipokines, such as adiponectin, exert protective metabolic effects. Together, these pathways may contribute to the development of insulin resistance and prediabetes.

Several indices have been proposed to identify individuals at increased metabolic risk. Among these, markers such as the Homeostatic Model Assessment for Insulin Resistance (HOMA-IR) and the Quantitative Insulin Sensitivity Check Index (QUICKI) are widely used to estimate insulin resistance and insulin sensitivity. However, both require insulin measurements, and their applicability is limited in routine clinical practice and large epidemiological studies. In recent years, increasing attention has been directed toward biomarkers derived from routine biochemical parameters that may better reflect the complex metabolic alterations associated with early dysglycemia.

Among these markers, the uric acid-to-high-density lipoprotein cholesterol ratio (UHR) has emerged as a potential indicator of cardiometabolic dysfunction. The biological rationale supporting this index is plausible and involves the opposing metabolic effects of its two components. Elevated serum uric acid levels have been associated with oxidative stress, endothelial dysfunction, inflammation, and impaired insulin signaling [6]. In contrast, HDL cholesterol exerts antioxidant and anti-inflammatory effects and plays an important role in reverse cholesterol transport [5] and endothelial homeostasis [7]. Therefore, UHR may integrate pro-inflammatory and protective metabolic pathways associated with glucose dysregulation.

Previous studies have reported associations between UHR and obesity, metabolic syndrome [8], insulin resistance [8,9], type 2 diabetes [10], and non-alcoholic fatty liver disease [11,12]. Moreover, some reports have suggested that elevated UHR values are associated with higher odds of prediabetes and impaired glucose metabolism [3]. Nevertheless, evidence specifically evaluating this relationship remains limited, particularly in Hispanic populations. Considering the high prevalence of prediabetes and diabetes in Mexico [13], identifying accessible biomarkers associated with early metabolic dysfunction could have clinical and epidemiological relevance. Therefore, the aim of this study was to evaluate the association between UHR and prediabetes in a cohort of Mexican-Mestizo adults from the Genetics of Atherosclerotic Disease (GEA) study.

## 2. Materials and Methods

### 2.1. Study Design and Patients

This study is a sub-analysis of the Genetics of Atherosclerotic Disease (GEA) study. Complete methods and preliminary results have been published elsewhere [14]. Briefly, the GEA study was developed to explore the genetic determinants of coronary artery disease (CAD) and to assess the association between both traditional and emerging cardiovascular risk factors in the Mexican population. All the data were obtained from validated sociodemographic, clinical and lifestyle habits questionnaires applied for the GEA study. Participants included men and women older than 20 years recruited from blood donors at the Instituto Nacional de Cardiología Ignacio Chávez, as well as individuals invited from community health clinics across Mexico City’s metropolitan area [14]. For the present analysis, we included 1395 participants (87% of the original cohort) who were recruited between 2008 and 2012 and had no evidence of coronary artery disease. Subjects with impaired fasting glucose (100 to 125 mg/dL) were considered with prediabetes. Participants were excluded if they had type 2 diabetes (defined as fasting blood glucose ≥ 126 mg/dL, use of glucose-lowering medication, or a previous diagnosis of diabetes) [15], clinical evidence of kidney disease (serum creatinine levels outside the normal reference range of 0.7–1.3 mg/dL in men and 0.5–1.1 mg/dL in women), or liver disease (viral or drug-induced hepatitis). The study protocol (No. 09-646) was approved by the National Institute of Cardiology Ethics Committee. All participants signed an informed consent letter.

### 2.2. Exposure, Outcome and Covariate Definitions

The exposure variable was the UHR, which was calculated as serum uric acid divided by HDL-C levels (mg/dL) measured in fasting serum. We stratified the population into sex-specific tertiles (T) of UHR, whose cutoff values were defined as follows: lowest tertile (T1 < 0.13 for men, <0.08 for women), middle tertile (T2: ≥0.13 to <0.18 and ≥0.08 to <0.11 for men and women respectively) and upper tertile (T3: ≥0.18 for men, ≥0.11 for women). Additionally, to further characterize the population, baseline characteristics were compared by glycemic status, stratifying participants into prediabetes.

### 2.3. Clinical Variables

For this sub-analysis, we extracted key variables from the GEA study and classified them into anthropometric, clinical and biochemical measurements. Our clinical variables were sex, age, body mass index (BMI), physical activity, active smoking, alcohol intake, systolic (SBP) and diastolic blood pressure (DBP), arterial hypertension (defined as a SBP ≥ 140 mmHg or DBP ≥ 90 mmHg and/or use of antihypertensive drugs) [16], having any dyslipidemia (defined as having total cholesterol ≥ 200 mg/dL, LDL-C ≥ 100 mg/dL, triglycerides ≥ 150 mg/dL) and/or HDL-C < 40 mg/dL in men and <50 mg/dL in women, metabolic syndrome (based on the NCEP-ATP III criteria) [17] and hyperuricemia (defined as serum uric acid levels > 7 mg/dL in men, and >6 mg/dL in women) [18]. Biochemical assessment included triglycerides, total cholesterol, LDL-C, HDL-C, non-HDL-C, uric acid, Apo-AI, Apoprotein B (Apo-B) and hsCRP. Adipose tissue markers included BMI categories (normal weight as 18.5–24.9 kg/m^2^, overweight as 25–29.9 kg/m^2^ and obesity as ≥30 kg/m^2^), insulin resistance (estimated through a HOMA-IR greater than 3.44 and 3.45 in men and women [≥75th percentile], respectively) [19] and adiponectin serum levels. Total, visceral and subcutaneous adipose tissue were measured through non-contrasted computed tomography (Somatom Sensation 64, Siemens Healthineers, Erlangen, Germany). Increased total, visceral and subcutaneous adipose tissue were defined as levels above the 75th percentile for each of them. We used the following thresholds: 152.5 cm^2^ in men and 121 cm^2^ in women for non-contrasted computed tomography visceral adipose tissue (VAT) and 221 cm^2^ in men and 320.5 cm^2^ in women for non-contrasted computed tomography subcutaneous adipose fat (SAT). In order to measure the non-contrasted computed tomography total adipose tissue (TAT), VAT and SAT, the methods described by Kvist H. et al. were used [20]. Inflammatory cytokines, including interleukin-6 (IL-6) and tumor necrosis factor-alpha (TNF-α), were measured in fasting serum samples using a commercially available multiplex bead-based assay (Luminex Discovery Assay, R&D Systems/Bio-Techne, Minneapolis, MN, USA) following the manufacturer’s protocol.

### 2.4. Statistical Analysis

The distribution of continuous variables was determined using the Kolmogorov–Smirnov test. Given the non-normal distribution of the data, continuous variables were expressed as median and interquartile range (IQR) and categorical variables as frequency and absolute percentages. To compare descriptive characteristics across UHR tertiles and prediabetes status, we used the Kruskal–Wallis and the Mann–Whitney U test for continuous variables, and the X^2^ test for categorical variables. All statistical analyses and creation of figures were performed in R Studio (Version 4.4.3; R Foundation for Statistical Computing, Vienna, Austria).

### 2.5. Cardiometabolic Profile, Association of UHR and Outcomes

First, we conducted a comparison of key cardiometabolic comorbidities by prediabetes status to determine if individuals with prediabetes exhibited an adverse cardiometabolic profile. For this descriptive analysis, we included hypertriglyceridemia (>150 mg/dL), metabolic syndrome, overweight/obesity, arterial hypertension, insulin resistance, increased TAT and LDL-C > 100 mg/dL. The results were reported in percentage and 95% CI estimated through the using the Clopper–Pearson interval method. Then, we fitted binomial logistic regression models to evaluate the association between the UHR and prediabetes. The UHR was multiplied by 10 in order to estimate the effect per 1 decile of increase in the ratio. Additionally, these models were conducted across tertiles of the UHR, taking the lower tertile as reference. Two models were fitted per UHR exposure evaluation (per 1 decile increase and per tertile). An unadjusted model (Model 1) and an adjusted model (Model 2) fitted for confounders. Confounders were selected from a priori direct acyclic graphs (DAGs) to identify the minimal set needed to block non-causal pathways between UHR and prediabetes. [21]. Based on the DAGs presented in Supplementary Figure S1, we considered age, sex, BMI, alcohol consumption, active smoking, arterial hypertension, and physical activity as potential confounders. For each model, we extracted the odds ratio (OR) with the 95% confidence intervals (95% CIs). Model diagnostics were performed in order to evaluate the assumptions of the logistic regression analyses.

Finally, we conducted sensitivity analyses to estimate the association for each individual predictor of the UHR index (uric acid, HDL-C and uric acid plus HDL-C) against prediabetes. We fitted non-adjusted binomial logistic regression models with each individual predictor and extracted the predicted probabilities to assess ROC curve analyses. Then we determine the sensitivity of the UHR in detecting prediabetes, as well as for their components using the Youden method. Receiver operating characteristic (ROC) curve analyses were performed to evaluate the ability of UHR, uric acid, HDL-C, HOMA-IR, fasting insulin, and Adipo-IR to identify prediabetes. Optimal cutoff values were determined using the Youden index. To formally compare the discriminatory performance among biomarkers, pairwise comparisons of AUROC values were conducted using DeLong’s nonparametric test for correlated ROC curves.

### 2.6. Metabolic Pathway Analysis

To explore potential biological pathways associated with UHR, prediabetes and adverse cardiometabolic traits, an exploratory in silico pathway analysis was performed using ShinyGO v0.85 (South Dakota State University, Brookings, SD, USA) [22]. A predefined set of genes involved in the principal inflammatory and metabolic processes evaluated in this study was selected, including *IL6*, *TNF*, *ADIPOQ*, *INS*, *IRS1*, *IRS2*, *NFKB1*, and *NFKB2*. These genes were chosen because they are directly related to inflammation, adipose tissue dysfunction, insulin signaling, and metabolic regulation, which represent biological pathways potentially related to the observed association between UHR and prediabetes.

Functional enrichment analysis was conducted using the Kyoto Encyclopedia of Genes and Genomes (KEGG) database (KEGG, Kanehisa Laboratories, Kyoto University, Kyoto, Japan) [23] implemented in ShinyGO. Since uric acid and HDL-C are metabolites rather than gene products, they were represented indirectly through genes involved in metabolic and inflammatory pathways associated with these biomarkers. Multiple testing correction was performed using the false discovery rate (FDR) provided by ShinyGO.

## 3. Results

### 3.1. Baseline Characteristics

**Figure 1:**
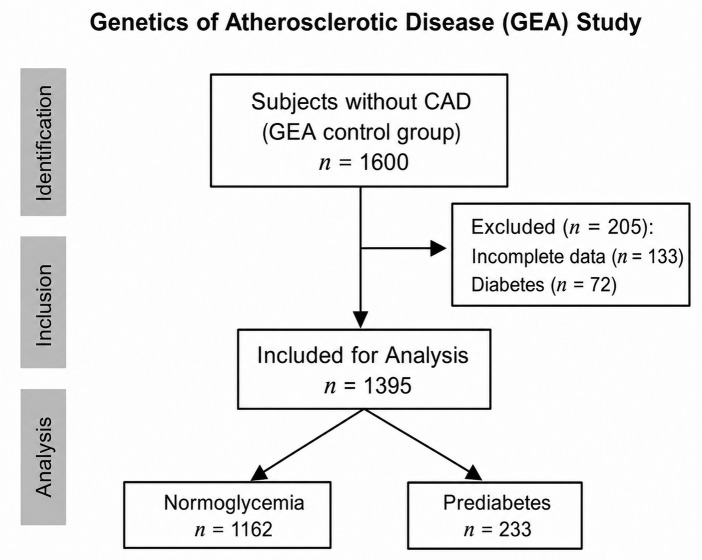
Flow-chart of the studied sample. Diagram constructed following the STROBE (Strengthening the Reporting of the Observational Studies in Epidemiology) guidelines. CAD, coronary artery disease.

From the original recruitment of the GEA control cohort (*n* = 1600) we excluded participants with incomplete data (*n* = 133, for both sexes, at least one of the following data: TG, HDL-C, uric acid, or HOMA-IR cannot be quantified (*n* = 70 women and 63 men) and participants living with diabetes (*n* = 72)), resulting in a studied sample of 1395 (87%) individuals (Figure 1).

The baseline characteristics of the studied participants stratified by UHR tertiles are presented in Table 1. Briefly, our sample comprised 51% (*n* = 708) women. The overall mean age was 53 ± 6 years.

Regarding the overall sample anthropometric measurements, the median BMI of the sample was 28.0 (IQR: 25.5–31.0), for SBP it was 114 (IQR: 105–125) and for DBP it was 72 (IQR: 66–78), while the median of physical activity was 7.88 (IQR: 7.00–8.75). The medians of uric acid and HDL-C levels were 5.54 (IQR: 4.59–6.60) and 45 (IQR: 36–54), respectively. Additionally, we found that 22% (*n* = 311) of the studied sample had active smoking, 75% (*n* = 1052) had positive alcohol intake, 23% (*n* = 326) had arterial hypertension, 28% (*n* = 393) had any dyslipidemia and 39% (*n* = 548) had metabolic syndrome. Most of these concomitant comorbidities were concentrated at the highest tertile of UHR. In relation to the biochemical assessment, participants in the highest UHR tertile exhibited higher levels of triglycerides, Apo-B, and hsCRP, together with lower concentrations of HDL-C, Apo-AI, and HDL particle diameter. Furthermore, individuals in the highest tertile showed a more adverse adipose tissue profile characterized by higher HOMA-IR values and greater adipose tissue accumulation across all compartments (VAT, SAT, and TAT), as well as lower adiponectin concentrations. Regarding glycemic status, 17% of the studied population had prediabetes. Notably, the prevalence of prediabetes progressively increased across UHR tertiles, from 11% in the lowest tertile to 15% in the middle tertile and 24% in the highest tertile.

### 3.2. Clinical and Metabolic Characteristics According to Glycemic Status

Table 2 shows the studied sample stratified by glycemic status; participants with prediabetes (*n* = 233) exhibited an adverse metabolic profile compared to those without this condition (*n* = 1162). Specifically, participants with prediabetes tended to be older and men. Regarding clinical profile, participants with prediabetes not only showed higher median values for BMI, SBP and DBP, but also a higher prevalence of arterial hypertension, dyslipidemia and metabolic syndrome. Medication use was evaluated since drugs may affect uric acid, HDL-C, glucose metabolism, and blood pressure. However, three medications showed significant use rates: beta blockers (normoglycemic *n* = 48 (4.1%) vs. prediabetic *n* = 15 (6.4%)), Angiotensin II antagonists (normoglycemic *n* = 41 (3.2%) vs. prediabetic *n* = 16 (6.7%)), and Diuretics (normoglycemic *n* = 34 (2.9%) vs. prediabetic *n* = 15 (6.4%)), *p* = 0.04, 0.003, and 0.001, respectively. Additional adjustment for these medications in the multivariable model did not materially change the observed associations between UHR and prediabetes.

Lower median values for physical activity were seen in the participants with prediabetes, whereas active smoking was higher among this group. Additionally, participants with prediabetes showed higher levels of triglycerides, non-HDL-C, uric acid, UHR, Apo-B and hsCRP, while lower concentrations of HDL-C, Apo-AI and HDL diameter were seen in this group. The median UHR was 0.12 (IQR: 0.09–0.17).

Given the clear differences in adiposity, biochemical, and metabolic indices between women and men, we analyze these variables in subjects with normal and impaired glucose tolerance. As shown in Table 3, the BMI, HOMA-IR, and adiponectin were similar in women and men with normal glucose levels. In women, the prevalence of VAT and SAT, as measured by non-contrasted tomography, was lower, even though the OB determined by waist circumference was higher. The mean FFA and HDL-C levels, and the prevalence of OB, were higher in women than in men (*p* < 0.001). Meanwhile, uric acid and UHR were significantly lower. A similar pattern was observed in the prediabetic group; however, insulin, HOMA-IR, and Adipo-IR were higher in prediabetic women than in normoglycemic women. In the prediabetic group, the prevalence of menopause was higher (74% vs. 64%). Adiponectin levels were similar in both the normoglycemic and impaired glucose groups.

### 3.3. Association of UHR and Prediabetes

To ensure model stability and avoid potential redundancy among adiposity-related variables, correlation analyses and variance inflation factor (VIF) diagnostics were performed before defining the final adjustment model. Moderate-to-strong correlations were observed among BMI, VAT, SAT, and TAT, particularly between SAT and TAT (r = 0.909) and between BMI and TAT (r = 0.830) (Supplementary Table S1). Therefore, variables showing substantial overlap were carefully evaluated to avoid introducing redundant information into the regression models. Moreover, model diagnostics indicated adequate model performance. The Hosmer–Lemeshow goodness-of-fit test showed no evidence of poor calibration (χ^2^ = 7.95, df = 8, *p* = 0.438). The model explained a modest proportion of outcome variability (Nagelkerke R^2^ = 0.114; McFadden R^2^ = 0.077).

The final adjusted model retained variables with low collinearity, all presenting VIF values below 2 (Supplementary Figure S2), indicating adequate model stability and absence of problematic multicollinearity. Accordingly, the final model was adjusted for age, sex, BMI, smoking status, alcohol consumption, hypertension, and physical activity.

After accounting for potential confounders, higher UHR remained independently associated with greater odds of prediabetes. Specifically, each 0.1-unit increase in UHR was associated with 65% higher odds of prediabetes (OR 1.65, 95% CI 1.26–2.17, *p* < 0.001). In the tertile analysis, participants in the middle UHR tertile had 1.25-fold higher odds of prediabetes (OR 1.25, 95% CI 0.82–1.90, *p* = 0.304), whereas those in the highest tertile had more than two-fold higher odds of prediabetes (OR 2.03, 95% CI 1.36–3.05, *p* < 0.001) compared with individuals in the lowest tertile (Table 4).

To further investigate the sex-specific differences observed in the predictive performance analyses, a formal interaction between UHR and sex was evaluated in the logistic regression model. The inclusion of the interaction term significantly improved model fit compared with the main-effects model (likelihood-ratio χ^2^ = 13.58, *p* < 0.001). Model calibration remained adequate after inclusion of the interaction term (Hosmer–Lemeshow χ^2^ = 9.62, df = 8, *p* = 0.293), and the explanatory capacity of the model increased (Nagelkerke R^2^ = 0.131; McFadden R^2^ = 0.089).

A significant interaction was observed in both the unadjusted model (OR interaction = 0.29, 95% CI 0.17–0.49, *p* < 0.001) and the adjusted model (OR interaction = 0.35, 95% CI 0.20–0.61, *p* < 0.001), indicating that the association between UHR and prediabetes was modified by sex. In sex-specific analyses, UHR was strongly associated with prediabetes in women (adjusted OR 3.39, 95% CI 2.10–5.53, *p* < 0.001), whereas no significant association was observed in men (adjusted OR 1.19, 95% CI 0.84–1.67, *p* = 0.318) (Table 5).

### 3.4. Sensitivity Analyses

**Figure 2:**
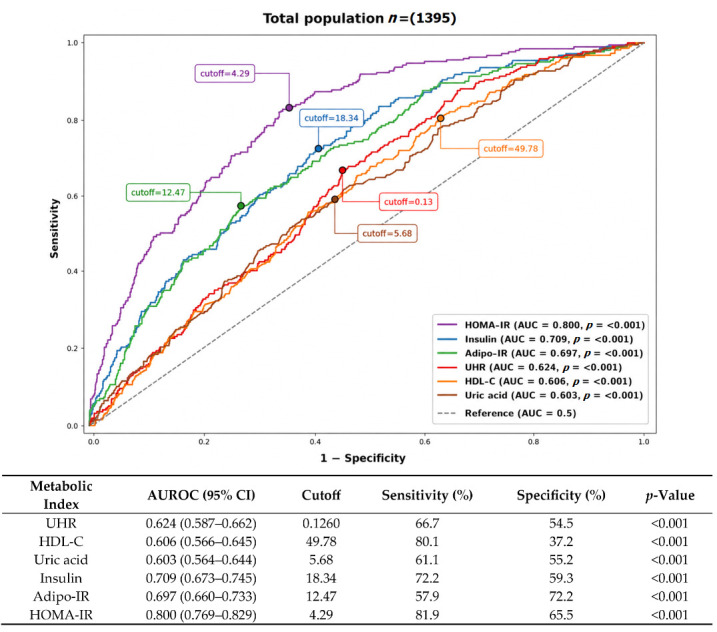
Receiver operating characteristic (ROC) curves for the prediction of prediabetes in the total population (*n* = 1395). Abbreviations: HOMA-IR, Homeostatic Model Assessment of Insulin Resistance; Adipo-IR, adipose tissue insulin resistance; UHR, uric acid-to-HDL cholesterol ratio; HDL-C, high-density lipoprotein cholesterol.

To further evaluate the discriminatory performance of the UHR for identifying prediabetes, we compared its diagnostic capacity against established metabolic indices associated with insulin resistance and adipose tissue dysfunction using receiver operating characteristic (ROC) curve analyses (Figure 2). Specifically, we compared UHR with HOMA-IR, a widely used marker of hepatic insulin resistance; fasting insulin levels; and Adipo-IR, an index that reflects adipose tissue insulin resistance.

Among the evaluated markers, HOMA-IR showed the highest discriminatory performance, with an area under the ROC curve (AUROC) of 0.80 (95% CI 0.74–0.86), followed by insulin levels (AUROC 0.709, 95% CI 0.64–0.78) and Adipo-IR (AUROC 0.697, 95% CI 0.63–0.77). The UHR showed a modest but significant discriminatory capacity, with an AUROC of 0.624 (95% CI 0.55–0.70).

Using cutoff values derived from the Youden index, HOMA-IR showed a sensitivity of 81.9% and specificity of 65.4%, whereas insulin levels showed a sensitivity of 72.2% and specificity of 59.3%. Adipo-IR exhibited a sensitivity of 57.9% and specificity of 72.2%. For UHR, the Youden-derived cutoff value was 0.13, with a sensitivity of 66.7% and specificity of 54.5%. These thresholds should be considered exploratory and applicable only to the present study population. Additional studies including internal and external validation, calibration assessment, and evaluation of clinical utility are required before any cutoff can be considered for clinical implementation.

UHR remained significantly associated with prediabetes and may represent a simple and accessible complementary biomarker derived from routine biochemical measurements. However, the discriminatory performance and cutoff values observed in this study require validation in independent populations before clinical application.

**Figure 3:**
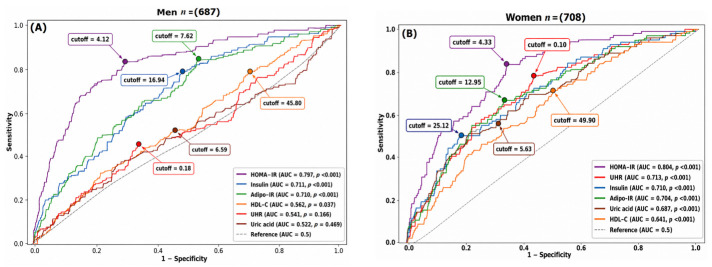
Receiver operating characteristic (ROC) curves for the prediction of prediabetes stratified by sex. Panel (**A**) shows results for men (*n* = 687) and panel (**B**) for women (*n* = 708). Abbreviations: HOMA-IR, Homeostatic Model Assessment of Insulin Resistance; UHR, uric acid-to-HDL cholesterol ratio; Adipo-IR, adipose tissue insulin resistance; HDL-C, high-density lipoprotein cholesterol.

Given the well-established sex-related differences in adiposity distribution, insulin sensitivity, lipid metabolism, and serum uric acid levels, we additionally performed ROC curve analyses stratified by sex to further evaluate the discriminatory performance of UHR and other metabolic indices for identifying prediabetes (Figure 3 and Table 6).

In both women and men, HOMA-IR exhibited the highest discriminatory performance, followed by fasting insulin levels and Adipo-IR. Although the discriminatory capacity of UHR was lower compared with insulin resistance-related indices in both sexes, UHR maintained a consistent performance pattern between women and men. Importantly, while HOMA-IR and Adipo-IR require insulin measurements and additional calculations, UHR can be derived from routine biochemical parameters that are readily available in clinical practice. Overall, these findings suggest that UHR may represent a simple and accessible complementary marker associated with prediabetes in both sexes, particularly in settings where direct insulin measurements are not routinely available.

To further compare the discriminatory performance of UHR against its individual components and established insulin resistance-related indices, DeLong tests were performed to compare the areas under the ROC curves (AUROCs). In the overall population, HOMA-IR, insulin, and Adipo-IR showed significantly higher AUROC values than UHR (all *p* < 0.01), whereas no significant differences were observed between UHR and either HDL-C or uric acid alone. In sex-stratified analyses, UHR demonstrated a discriminatory performance comparable to insulin and Adipo-IR among women (all *p* > 0.05) and significantly greater performance than HDL-C alone (*p* < 0.001). Conversely, in men, UHR showed significantly lower AUROC values compared with HOMA-IR, insulin, and Adipo-IR (all *p* < 0.001), while no differences were observed when compared with HDL-C or uric acid. These findings suggest that the combination of uric acid and HDL-C into a single index may provide additional discriminatory information beyond either biomarker alone (Supplementary Table S2).

## 4. Discussion

In this cohort of Mexican-Mestizo adults, higher UHR was independently associated with prediabetes, with a clear dose–response pattern across tertiles. Individuals in the highest UHR tertile had approximately two-fold higher odds of prediabetes compared with those in the lowest tertile. These findings suggest that elevated UHR may capture metabolic alterations associated with early glucose dysregulation in this population [7].

The adverse cardiometabolic profile associated with elevated UHR was evident from the baseline analyses. Participants in the highest UHR tertile exhibited higher BMI, blood pressure, triglycerides, Apo-B, hsCRP, HOMA-IR, and Adipo-IR values, together with greater visceral, subcutaneous, and total adipose tissue accumulation. In contrast, HDL-C, Apo-AI, and adiponectin concentrations progressively decreased across increasing UHR tertiles. Likewise, the prevalence of metabolic syndrome increased markedly from 14% in the lowest tertile to 68% in the highest, whereas prediabetes prevalence followed the same gradient (11%, 15%, and 24%, respectively). Altogether, these findings indicate that elevated UHR identifies individuals with a metabolically unhealthy phenotype characterized by adiposity [24], insulin resistance [25], systemic inflammation [26], and lipid abnormalities [27], consistent with previous reports linking UHR to obesity and hepatic and adipose tissue insulin resistance [24,28,29].

A similar metabolic profile was observed among individuals with prediabetes. Compared with normoglycemic participants, subjects with prediabetes were older, more likely to be men, and exhibited higher BMI and prevalence of hypertension, dyslipidemia, and metabolic syndrome. Biochemically, they showed higher triglycerides, non-HDL-C, uric acid, UHR, Apo-B, and hsCRP levels, together with lower HDL-C, Apo-AI, and adiponectin concentrations. HOMA-IR values were nearly twice as high in the prediabetes group, and all adipose tissue compartments were significantly larger. Similar elevations of UHR in prediabetic individuals have been reported in other populations, including a retrospective study conducted in Turkish individuals [5]. Therefore, validating these findings in Mexican-Mestizo adults was particularly relevant given the high burden of prediabetes and diabetes in Mexico [30,31].

After adjustment for potential confounders selected a priori through DAG-based methodology, each 0.1-unit increase in UHR was associated with 61% greater odds of prediabetes. Moreover, the progressive increase in odds across UHR tertiles is consistent with the possibility that the association is not limited to a simple threshold effect, but rather may represent a graded relationship between UHR and early dysglycemia. These findings are consistent with previous studies showing that UHR is positively associated not only with prediabetes, but also with obesity, insulin resistance, metabolic syndrome, and type 2 diabetes across different clinical settings [6,7,32]. Importantly, UHR is derived from routine biochemical measurements and may provide metabolic information not fully captured by traditional cardiometabolic risk factors.

**Figure 4:**
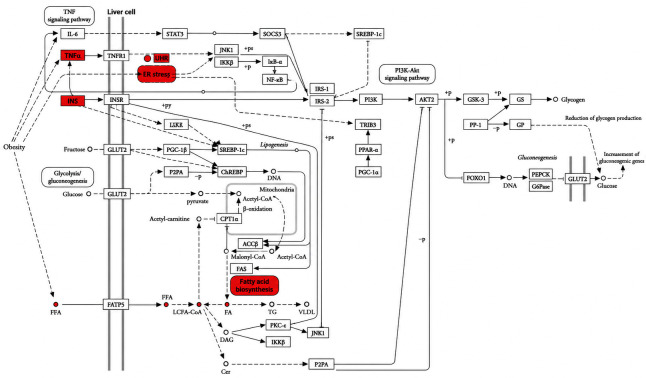
Metabolic and inflammatory pathway interactions potentially associated with elevated UHR and prediabetes. The biological framework was generated by integrating the principal findings of the present study with KEGG pathways identified through an exploratory ShinyGO pathway analysis based on the genes IL6, TNF, ADIPOQ, INS, IRS1, IRS2, NFKB1, and NFKB2. Elevated uric acid-to-high-density lipoprotein cholesterol ratio (UHR) may reflect biological processes related to adipose tissue dysfunction, chronic low-grade inflammation, insulin resistance, and lipid abnormalities that have been associated with early dysglycemia. The interactions illustrated in the figure are intended as a conceptual and hypothesis-generating framework derived from pathway enrichment analysis and existing biological knowledge. Therefore, the proposed relationships should not be interpreted as evidence of causality or direct mechanistic effects. The most significantly enriched KEGG pathway identified was type II diabetes mellitus (FDR = 1.2 × 10^−8^).

The relationship between UHR and prediabetes is likely multifactorial and may involve interconnected inflammatory and metabolic pathways [33]. Chronic low-grade inflammation derived from adipose tissue has been recognized as a central mechanism in the development of insulin resistance and glucose dysregulation [34]. The potential mechanisms linking elevated UHR with prediabetes and metabolic dysfunction are summarized in Figure 4. In this context, pro-inflammatory cytokines such as TNF-α may contribute to impaired insulin signaling and alterations in lipid metabolism [35], promoting adipose tissue dysfunction [36,37,38] and increased circulating free fatty acids [39,40]. In our study, individuals with elevated UHR also exhibited higher circulating TNF-α levels, further supporting the presence of a chronic low-grade inflammatory state associated with adipose tissue dysfunction and early glucose dysregulation. Elevated free fatty acid levels favor peripheral and hepatic insulin resistance [41], which was reflected in our study by the progressive increase in HOMA-IR and Adipo-IR across higher UHR categories [42]. In parallel, lower adiponectin concentrations observed in participants with elevated UHR may further contribute to metabolic dysfunction given the insulin-sensitizing and anti-inflammatory properties of this adipokine. Together, these findings support the concept that UHR may reflect an integrated metabolic-inflammatory state associated with early dysglycemia.

To further evaluate the discriminatory performance of UHR, we compared this index against established metabolic markers associated with insulin resistance and adipose tissue dysfunction. As expected, HOMA-IR, fasting insulin, and Adipo-IR showed superior discriminatory capacity for identifying prediabetes. Nevertheless, UHR showed a broadly consistent discriminatory pattern across both sexes. Although its discriminatory capacity was lower than that of insulin resistance-related indices, UHR could represent a potentially useful practical alternative in some settings, given that it can be derived from routine biochemical measurements without requiring insulin determinations or specialized calculations. However, the discriminatory performance of UHR was modest (AUROC = 0.624), with a specificity of 54.5%. Therefore, despite its significant association with prediabetes, UHR should not be interpreted as a standalone screening or diagnostic tool. Rather, it may serve as a complementary marker of cardiometabolic dysfunction, particularly in settings where insulin measurements are not routinely available. Additionally, the discriminatory performance of UHR was slightly higher than that observed for either uric acid or HDL-C alone. The AUROC for UHR was 0.624 (95% CI 0.587–0.662), compared with 0.603 (95% CI 0.564–0.644) for uric acid and 0.606 (95% CI 0.566–0.645) for HDL-C (Figure 2), suggesting that the combined index may better capture the metabolic imbalance associated with prediabetes.

Previous work within the same GEA cohort identified UHR as a useful marker associated with non-alcoholic fatty liver disease [30,31], whereas other studies have linked elevated UHR values with NAFLD in non-obese individuals [43].

The biological rationale supporting UHR rests on the opposing physiological effects of its two components, both closely linked to oxidative stress and inflammation. Elevated uric acid levels promote oxidative stress [44], endothelial dysfunction, inflammatory cytokine production [45], and impaired insulin signaling, whereas HDL cholesterol exerts anti-inflammatory, antioxidant, and antiatherogenic effects [46]. Therefore, the balance between elevated uric acid and reduced HDL-C may reflect a metabolic environment favoring cardiometabolic dysfunction. In this regard, UHR emerges as a potential clinical biomarker because it is inexpensive, non-invasive, and easily applicable in routine clinical practice. This study has several strengths. First, we evaluated a well-characterized cohort with extensive clinical, biochemical, inflammatory, and adiposity-related phenotyping. Second, adipose tissue compartments were quantified using computed tomography, allowing a detailed characterization of body fat distribution. Third, we incorporated inflammatory and adipose tissue dysfunction markers, including TNF-α, adiponectin, HOMA-IR, and Adipo-IR, which allowed a more comprehensive evaluation of the metabolic alterations associated with elevated UHR. Finally, evidence regarding UHR and prediabetes in Hispanic populations remains limited, making our findings particularly relevant for the Mexican and Hispanic population.

Higher UHR was independently associated with prediabetes and with an adverse metabolic-inflammatory profile in Mexican-Mestizo adults. Elevated UHR values were associated with greater adiposity, insulin resistance, inflammatory markers, and lipid abnormalities, supporting its role as a complementary marker of early metabolic dysfunction. Further longitudinal studies are needed to determine whether UHR may help identify individuals with probability of developing type 2 diabetes and related cardiometabolic complications.

## 5. Limitations

Some limitations of this study should be acknowledged. First, due to the retrospective cross-sectional design, causal relationships and temporal associations between UHR and prediabetes cannot be established. Therefore, it is not possible to determine whether elevated UHR precedes the development of prediabetes or reflects metabolic alterations already present during early dysglycemia. This study consisted of a Mexican sample population and may not have represented the entire Latino population. Additionally, prediabetes was defined exclusively using fasting plasma glucose levels because HbA1c measurements and oral glucose tolerance test data were unavailable in the GEA cohort. Consequently, some individuals with prediabetes may have been misclassified as normoglycemic, and the true prevalence of prediabetes may have been underestimated.

Another limitation of this work is that since the sample consisted of volunteers, participants may not have represented the general population. Nevertheless, the prevalence of coronary heart disease risk factors observed is similar to that found in the ENSANUT, a survey with national representation [47]. The findings have not been externally validated in independent populations. Therefore, replication studies in other cohorts are necessary to confirm the robustness and reproducibility of the observed associations.

Finally, the pathway analysis was based on an in silico approach using predefined genes related to the principal inflammatory and metabolic biomarkers evaluated in this study. Therefore, the identified pathways represent biologically plausible mechanisms rather than experimentally validated molecular interactions. Future studies incorporating transcriptomic, proteomic, or functional analyses are needed to confirm the involvement of these pathways in the association between UHR and prediabetes.

## 6. Conclusions

In conclusion, higher UHR was associated with prediabetes and adverse metabolic profile in adults. Elevated UHR values were also linked to greater adiposity, insulin resistance, systemic inflammation, and lipid abnormalities, suggesting that this index may serve as a complementary marker of early metabolic dysfunction such as prediabetic status. Notably, the association between UHR and prediabetes was significantly stronger in women than in men, suggesting potential sex-specific differences in the relationship between UHR and early glucose dysregulation. Although its discriminatory performance was modest, UHR may represent a simple and accessible biomarker derived from routine laboratory measurements. However, it should not be considered a standalone screening or diagnostic tool for prediabetes. However, further longitudinal studies are needed to clarify whether elevated UHR precedes the development of type 2 diabetes and related cardiometabolic complications, and to better establish its predictive value.

## Figures and Tables

**Table 1:** Baseline study cohort characteristics stratified by tertiles of the UHR.

Characteristic	Overall Sample (*n* = 1395)	Lower Tertile (T1) * (*n* = 461)	Middle Tertile (T2) * (*n* = 458)	Upper Tertile (T3) * (*n* = 476)	*p*-Value
Clinical variables					
Sex					
Women *n* (%)	708 (51)	234 (51)	233 (51)	241 (51)	0.9
Men *n* (%)	687 (49)	227 (49)	225 (49)	235 (49)	
Age (years)	53 (47, 59)	54 (48, 59)	54 (47, 60)	52 (45, 58)	0.002
BMI (kg/m^2^)	28.0 (25.5, 31.0)	26.0 (23.8, 28.5)	28.2 (25.7, 31.0)	29.7 (27.4, 32.4)	<0.001
SBP (mmHg)	114 (105, 125)	114 (105, 125)	113 (104, 124)	115 (107, 127)	0.020
DBP (mmHg)	72 (66, 78)	71 (65, 77)	71 (66, 77)	73 (67, 79)	0.001
Physical activity ^a^	7.88 (7.00, 8.75)	8.00 (7.13, 8.88)	7.88 (7.00, 8.75)	7.75 (6.75, 8.50)	0.006
Active smoking *n* (%)	311 (22)	93 (20)	101 (22)	117 (25)	0.048
Alcohol intake *n* (%) ^b^	1052 (75)	363 (79)	357 (78)	332 (70)	0.002
Arterial hypertension *n* (%)	326 (23)	88 (19)	108 (24)	130 (27)	0.012
Dyslipidemia *n* (%) ^c^	393 (28)	117 (26)	122 (27)	154 (32)	0.008
MS *n* (%) ^d^	548 (39)	64 (14)	161 (35)	323 (68)	<0.001
Prediabetes *n* (%)	233 (17)	50 (11)	68 (15)	115 (24)	<0.001
Biochemical assessment					
Triglycerides (mg/dL)	146 (110, 201)	117 (88, 147)	148 (112, 191)	189 (144, 255)	<0.001
Total cholesterol (mg/dL)	192 (168, 213)	193 (169, 216)	192 (171, 213)	190 (163, 210)	0.082
LDL-C (mg/dL)	118 (97, 138)	114 (94, 134)	122 (102, 139)	117 (97, 138)	0.003
HDL-C (mg/dL)	45 (36, 54)	56 (49, 66)	44 (39, 51)	35 (30, 41)	<0.001
Uric acid (mg/dL)	5.54 (4.59, 6.60)	4.57 (3.84, 5.39)	5.53 (4.83, 6.37)	6.60 (5.68, 7.71)	<0.001
Apo-AI (mg/dL)	133 (115, 157)	151 (132, 171)	131 (116, 155)	119 (104, 137)	<0.001
Apo-B (mg/dL)	95 (77, 114)	87 (72, 106)	95 (80, 115)	100 (82, 119)	<0.001
hsCRP (mg/L)	1.56 (0.81, 3.23)	1.09 (0.61, 2.28)	1.56 (0.83, 2.95)	2.18 (1.08, 4.08)	<0.001
Insulin UI/L	17.1 (12.4, 23.6)	14.1 (9.9, 18.9)	16.3 (12.5, 21.9)	21.3 (15.4, 27.8)	<0.001
FFA mmol/L	0.55 (0.43, 0.69)	0.57 (0.43, 0.71)	0.56 (0.44, 0.71)	0.55 (0.43, 0.68)	0.24
IL-6	0.87 (0.43, 1.8)	0.86 (0.44, 1.6)	0.86 (0.45, 1.8)	0.97(0.39, 1.93)	0.15
TNF-a	0.57 (0.04, 1.81)	0.53 (0.01, 1.5)	0.60 (0.01, 1.8)	0.64 (1.6, 2.1)	0.014
Metabolic markers/Adipose tissue markers					
HOMA-IR	3.78 (2.60, 5.38)	2.98 (2.13, 4.17)	3.64 (2.68, 5.12)	4.95 (3.39, 6.84)	<0.001
TyG	1.6 (1.2, 2.2)	1.3 (0.98, 1.6)	1.5 (1.2, 2.1)	2.0 (1.6, 2.8)	<0.001
Adipo-IR (FFA/Insulin)	6.4 (6.0, 14.0)	7.5 (4.9, 11.5)	9.1 (5.9, 13.5)	11.3 (7.7, 16.6)	<0.001
Adiponectin (µg/mL)	8 (5, 13)	10 (6, 16)	8 (5, 13)	6 (4, 9)	<0.001
TAT (cm^2^)	437 (350, 541)	389 (297, 480)	432 (357, 541)	490 (403, 585)	<0.001
VAT (cm^2^)	149 (109, 190)	120 (86, 168)	150 (111, 190)	167 (133, 210)	<0.001
SAT (cm^2^)	284 (212, 360)	254 (190, 320)	282 (221, 360)	312 (245, 395)	<0.001

* Tertiles assigned by sex (men and women, respectively): T1 (<0.13, <0.08), T2 (≥0.13 to <0.18, ≥0.08 to <0.11), T3 (≥0.18, ≥0.11). Variables are presented as *n* (%) or median (IQR). Annotations: (^a^) Physical activity was measured using the Baecke’s questionnaire. (^b^) Alcohol intake was any alcoholic drink within the last 6 months. (^c^) Dyslipidemia was defined by NCEP-ATP III criteria. (^d^) Metabolic syndrome was defined by NCEP-ATP III criteria. Abbreviations: UHR, uric acid-to-high-density lipoprotein cholesterol ratio, BMI, body mass index; SBP, systolic blood pressure; DBP, diastolic blood pressure; MS, metabolic syndrome; LDL-C, low-density lipoprotein cholesterol; HDL-C, high-density lipoprotein cholesterol; Apo, apoprotein; hsCRP, high-sensitivity C-reactive protein; HOMA-IR, Homeostatic Model Assessment for Insulin Resistance; TyG, triglycerides-to-glucose ratio; Adipo-IR, adipose tissue insulin resistance; TAT, non-contrasted computed tomography total adipose tissue; VAT, non-contrasted computed tomography visceral adipose tissue; SAT, non-contrasted computed tomography subcutaneous adipose tissue.

**Table 2:** Baseline study cohort characteristics stratified by glycemic status.

Characteristic	Overall Sample (*n* = 1395)	Normoglycemia (*n* = 1162)	Prediabetes (*n* = 233)	*p*-Value
Clinical variables				
Sex				
Women *n* (%)	708 (51)	603 (52)	105 (45)	0.057
Men *n* (%)	687 (49)	559 (48)	128 (55)	
Age (years)	53 (47, 59)	53 (46, 58)	55 (49, 62)	<0.001
BMI (kg/m^2^)	28.0 (25.5, 31.0)	27.6 (25.2, 30.5)	29.9 (27.3, 32.8)	<0.001
SBP (mmHg)	114 (105, 125)	113 (104, 124)	119 (110, 131)	<0.001
DBP (mmHg)	72 (66, 78)	71 (65, 77)	74 (69, 80)	<0.001
Physical activity ^a^	7.88 (7.00, 8.75)	7.88 (7.00, 8.75)	7.69 (6.75, 8.50)	0.048
Active smoking *n* (%)	311 (22)	268 (23)	43 (18)	0.123
Alcohol intake *n* (%) ^b^	1052 (75)	872 (75)	180 (77)	0.5
Arterial hypertension *n* (%)	326 (23)	244 (21)	82 (35)	<0.001
Dyslipidemia *n* (%) ^c^	393 (28)	313 (27)	80 (34)	0.037
MS *n* (%) ^d^	548 (39)	356 (31)	192 (82)	<0.001
Biochemical assessment				
Triglycerides (mg/dL)	146 (110, 201)	140 (105, 191)	173 (130, 236)	<0.001
Total cholesterol (mg/dL)	192 (168, 213)	191 (169, 211)	193 (168, 221)	0.3
LDL-C (mg/dL	118 (97, 138)	118 (98, 137)	117 (97, 139)	0.5
HDL-C (mg/dL)	45 (36, 54)	45 (37, 55)	41 (34, 48)	<0.001
Uric acid (mg/dL)	5.54 (4.59, 6.60)	5.46 (4.45, 6.50)	6.12 (5.07, 6.94)	<0.001
UHR	0.12 (0.09, 0.17)	0.12 (0.09, 0.17)	0.14 (0.11, 0.19)	<0.001
Apo-AI (mg/dL)	133 (115, 157)	134 (116, 158)	128 (112, 152)	0.023
Apo-B (mg/dL)	95 (77, 114)	94 (76, 113)	100 (82, 119)	0.003
hsCRP (mg/dL)	1.56 (0.81, 3.23)	1.46 (0.78, 3.03)	2.24 (1.01, 4.01)	<0.001
Insulin UI/L		16.1 (11.6, 22.1)	22.8 (17.2, 31.6)	<0.001
FFA mmol/L		0.55 (0.43, 0.69)	0.588 (0.45, 0.70)	0.055
IL6	0.87 (0.43, 1.8)	0.88 (0.44, 1.7)	0.84 (0.36, 1.87)	0.15
TNF-a	0.57 (0.04, 1.81)	0.55 (0.01, 1.7)	0.877(0.36, 2.26)	0.014
Metabolic markers/Adipose tissue markers				
HOMA-IR	3.78 (2.60, 5.38)	3.40 (2.47, 4.87)	6.11 (4.56, 8.26)	<0.001
TyG	1.6 (1.2, 2.2)	1.6 (1.2, 2.2)	1.6 (1.2, 2.2)	0.66
Adipo-IR (FFA/Insulin)	9.4 (6.0, 14.0)	8.8 (5.7, 13.0)	13.2 (8.8, 20.0)	<0.001
Adiponectin (ug/mL)	8 (5, 13)	8 (5, 13)	7 (4, 10)	<0.001
TAT (cm^2^)	437 (350, 541)	424 (339, 527)	492 (417, 590)	<0.001
VAT (cm^2^)	149 (109, 190)	142 (103, 183)	178 (140, 228)	<0.001
SAT (cm^2^)	284 (212, 360)	279 (207, 354)	306 (242, 390)	<0.001

Variables are presented as *n* (%) or median (IQR). Annotations: (^a^) Physical activity was measured using the Baecke’s questionnaire. (^b^) Alcohol intake was any alcoholic drink within the last 6 months. (^c^) Dyslipidemia was defined by NCEP-ATP III criteria. (^d^) Metabolic syndrome was defined by NCEP-ATP III criteria. Abbreviations: BMI, body mass index; SBP, systolic blood pressure; DBP, diastolic blood pressure; MS, metabolic syndrome; LDL-C, low-density lipoprotein cholesterol; HDL-C, high-density lipoprotein cholesterol; UHR, uric acid-to-high-density lipoprotein cholesterol ratio; Apo, apoprotein; hsCRP, high-sensitivity C-reactive protein; HOMA-IR, Homeostatic Model Assessment for Insulin Resistance; TyG, triglycerides-to-glucose ratio; Adipo-IR, adipose tissue insulin resistance; TAT, non-contrasted computed tomography total adipose tissue; VAT, non-contrasted computed tomography visceral adipose tissue; SAT, non-contrasted computed tomography subcutaneous adipose tissue.

**Table 3:** General characteristics by sex and glycemic status.

	Glucose <100 mg/dL			Glucose 100–125.9 mg/dL				
Variable	Women (*n* = 603)	Men (*n* = 559)	*p*	Women (*n* = 105)	Men (*n* = 128)	*p*	*p* *	*p* **
Age (years)	53 (47, 58)	52 (45, 59)	0.26	56 (50, 62)	54 (48, 62)	0.93	0.009	0.002
BMI (kg/m^2^)	27.4 (24.9, 30.6)	27.7 (25.4, 30.3)	0.26	31.1 (28.7, 33.8)	28.8 (26.8, 31.4)	<0.001	<0.001	0.002
Insulin (U/L)	16.3 (13.0, 22.2)	15.6 (11.2, 22.1)	0.89	23.5 (16.9, 32.1)	22.3 (17.2, 29.8)	0.68	<0.001	<0.001
HOMA-IR	3.4 (2.5, 4.9)	3.4 (2.4, 4.9)	0.88	6.3 (4.5, 8.4)	5.7 (4.5, 7.8)	0.56	<0.001	<0.001
FFA (mmol/L)	0.61 (0.49, 0.76)	0.49 (0.38, 0.60)	<0.001	0.64 (0.50, 0.79)	0.51 (0.43, 0.64)	<0.001	0.16	0.039
Adipo-IR	10.1 (6.7, 14.9)	7.7 (4.9, 11.4)	<0.001	15.9 (10.4, 21.5)	11.6 (8.1, 16.5)	0.021	<0.001	<0.001
Uric acid (mg/dL)	4.7 (4.0, 5.5)	6.3 (5.5, 7.2)	<0.001	5.7 (4.7, 6.4)	6.6 (5.5, 7.3)	<0.001	<0.001	0.363
HDL-C (mg/dL)	50.5 (42.0, 59.9)	40.0 (34.1, 48.0)	<0.001	44.4 (36.3, 53.1)	39.5 (33.0, 45.1)	<0.001	<0.001	0.054
UHR	0.09 (0.07, 0.12)	0.12 (0.10, 0.16)	<0.001	0.13 (0.10, 0.16)	0.16 (0.13, 0.21)	<0.001	<0.001	0.069
Adiponectin (µg/mL)	10.4 (6.6, 16.0)	6.2 (3.9, 9.6)	0.32	8.3 (5.1, 12.0)	5.5 (3.7, 9.2)	0.94	0.001	0.053
Obesity (%)	54.2	45.8	0.002	47.2	52.8	0.006		
VAT > p75 (%)	50.1	57.9	0.009	80.2	75.2	0.23		
SAT > p75 (%)	41.8	55.7	<0.001	66	69	0.36		
Menopause (%)	64.1	NA		74.5	NA			

BMI: body mass index; HOMA-IR: Homeostatic Model Assessment for Insulin Resistance; FFA: free fatty acids; Adipo-IR: adipose tissue insulin resistance; HDL-C: high-density lipoprotein cholesterol; VAT: visceral abdominal tissue; SAT: subcutaneous abdominal tissue; p75: 75th percentile; NA: not applicable. Data are presented as median (IQR) or percentage. *p*-value indicates comparison between women and men. *p* * between women and *p* ** between men with glucose < 100 mg/dL and glucose between 10 and 125.9 mg/dL.

**Table 4:** Logistic regression model for the association between the UHR and prediabetes.

		Unadjusted			Adjusted ^b^		
Exposure	Events *n* (%)	OR	95% CI	*p*	OR	95% CI	*p*
UHR (per 0.1 increase)	233 (17)	1.47	1.29–1.67	<0.001	1.65	1.26–2.17	<0.001
UHR by tertiles ^a^							
1	50 (11)	Reference			Reference		
2	68 (15)	1.43	0.97–2.13	0.071	1.25	0.82–1.90	0.304
3	115 (24)	2.62	1.84–3.78	<0.001	2.03	1.36–3.05	<0.001

Annotations: (^a^) Tertiles assigned by sex (men and women, respectively): T1 (<0.13, <0.08), T2 (≥0.13 to <0.18, ≥0.08 to <0.11), T3 (≥0.18, ≥0.11). (^b^) Models were adjusted for age, sex, BMI, active smoking, alcohol intake, arterial hypertension, and physical activity. Abbreviations: UHR, uric acid-to-high-density lipoprotein cholesterol ratio; OR, odds ratio; CI, confidence interval; BMI, body mass index.

**Table 5:** Sex-specific association between UHR and prediabetes and formal interaction analysis.

		Unadjusted			Adjusted ^a^		
Sex	Events *n* (%)	OR	95% CI	*p*-Value	OR	95% CI	*p*-Value
Interaction (UHR × sex)	—	0.29	0.17–0.49	<0.001	0.35	0.20–0.61	<0.001
Women	105 (14.8)	4.46	2.91–6.94	<0.001	3.39	2.10–5.53	<0.001
Men	128 (18.6)	1.31	0.97–1.76	0.070	1.19	0.84–1.67	0.318

Annotations: (^a^) Models were adjusted for age, BMI, active smoking, alcohol intake, arterial hypertension, and physical activity. Abbreviations: UHR, uric acid-to-high-density lipoprotein cholesterol ratio; OR, odds ratio; CI, confidence interval; BMI, body mass index.

**Table 6:** ROC analysis for the identification of prediabetes according to sex.

Men *n* (687)					
Metabolic Index	AUROC (95% CI)	Cutoff	Sensitivity (%)	Specificity (%)	*p*-Value
UHR	0.541 (0.481–0.600)	0.1767	46.1	63.7	0.166
HDL-C	0.562 (0.505–0.621)	45.8	80.9	31.4	0.037
Uric acid	0.522 (0.459–0.580)	6.59	51.3	56.8	0.469
Insulin	0.711 (0.660–0.757)	16.94	80.0	54.2	<0.001
Adipo-IR	0.710 (0.658–0.760)	7.62	84.3	48.9	<0.001
HOMA-IR	0.797 (0.754–0.838)	4.12	84.3	62.9	<0.001
**Woman *n* (708)**					
**Metabolic** **Index**	**AUROC (95% CI)**	**Cutoff**	**Sensitivity (%)**	**Specificity (%)**	***p*-Value**
UHR	0.713 (0.658–0.766)	0.1005	77.2	57.2	<0.001
HDL-C	0.641 (0.581–0.696)	49.9	70.3	51.8	<0.001
Uric acid	0.687 (0.630–0.745)	5.63	50.5	79.4	<0.001
Insulin	0.710 (0.655–0.762)	25.12	49.5	82.8	<0.001
Adipo-IR	0.704 (0.647–0.754)	12.95	66.3	67.1	<0.001
HOMA-IR	0.804 (0.758–0.844)	4.33	83.2	66.2	<0.001

Data are presented as area under the receiver operating characteristic curve (AUROC) with 95% confidence intervals, optimal cutoff values determined by the Youden index, sensitivity, specificity, and *p*-values. UHR, uric acid-to-HDL cholesterol ratio; HDL-C, high-density lipoprotein cholesterol; Adipo-IR, adipose tissue insulin resistance index; HOMA-IR, homeostatic model assessment of insulin resistance.

## Data Availability

Due to confidentiality agreements, the data underlying this study are not publicly available. Access to the data can be requested through gccardosos@yahoo.com following their confidentiality protocols.

## Supplementary Materials

The following supporting information can be downloaded at https://www.mdpi.com/article/10.3390/diagnostics16132038/s1. Figure S1: Direct acyclic graph to evaluate potential confounders and mediators related to the association between the UHR and the presence of prediabetes; Table S1: Correlation matrix among BMI VAT SAT and TAT; Figure S2: Uric acid to high-density lipoprotein cholesterol ratio (UHR) model assumption check: Diagnostic plots evaluating the predictive model for prediabetes; Table S2: DeLong test comparisons between UHR and other metabolic indices for the identification of prediabetes.
