# Supplementary material for: Uric Acid-to-HDL Cholesterol Ratio Is Associated with Prediabetes and an Adverse Metabolic Profile: Results from the Genetics of Atherosclerotic Disease (GEA) Study
Source: Diagnostics (Basel). 2026 Jun 30;16(13):2038. doi: 10.3390/diagnostics16132038 (PMC13360160; doi:10.3390/diagnostics16132038)
Supplement: Supplementary file 1 [file diagnostics-16-02038-s001.zip › diagnostics-4373368-supplementary.pdf]

Supplementary Figure S1: Direct acyclic graph to evaluate potential confounders and mediators related to the association between the UHR and the presence of prediabetes.

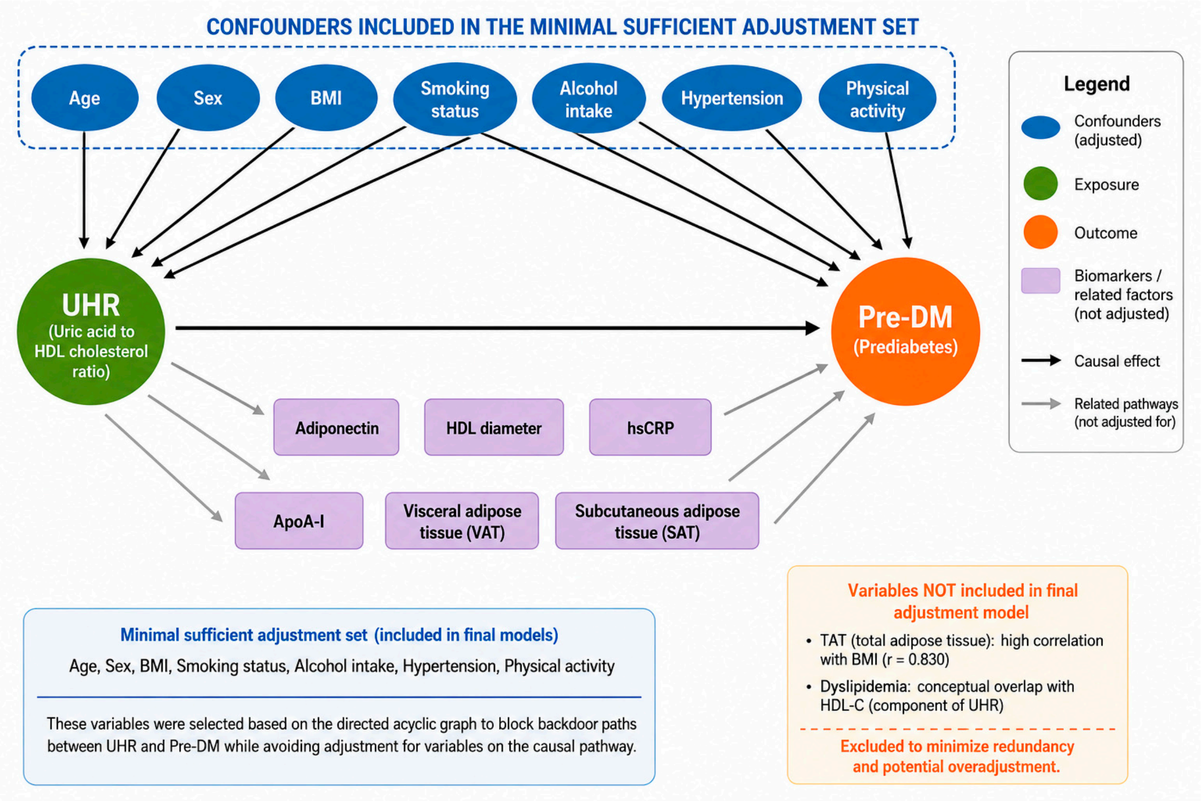

Abbreviations: UHR, UA/HDL-C Ratio; CT-TAT, computed tomography total abdominal tissue; CT-VAT, computed tomography visceral abdominal tissue; CT-SAT, computed tomography subcutaneous abdominal tissue; hsCRP, high sensitivity C-reactive protein; Apo, apolipoprotein; Pre-DM, prediabetes.

Supplementary Table S1. Correlation matrix among BMI VAT SAT and TAT

Variable	BMI	VAT	SAT	TAT
BMI	1.000	0.533	0.774	0.830
VAT	0.533	1.000	0.317	0.683
SAT	0.774	0.317	1.000	0.909
TAT	0.830	0.683	0.909	1.000

Abbreviations: BMI, body mass index; VAT, visceral adipose tissue; SAT, subcutaneous adipose tissue; TAT, total adipose tissue.

Supplementary Figure S2: Uric acid to high-density lipoprotein cholesterol ratio (UHR) model assumption check: Diagnostic plots evaluating the predictive model for prediabetes. (Top left) Posterior predictive check showing observed and model-predicted data. (Top right) Binned residuals analysis, where points within error bounds indicate a good fit. (Middle left) Influential observations assessed using standardized residuals vs. leverage. (Middle right) Collinearity diagnostics using the variance inflation factor (VIF), indicating acceptance levels (VIF <5). (Bottom left) Uniformity of residuals assessed by quantile-quantile (Q-Q) plot against the standard uniform distribution.

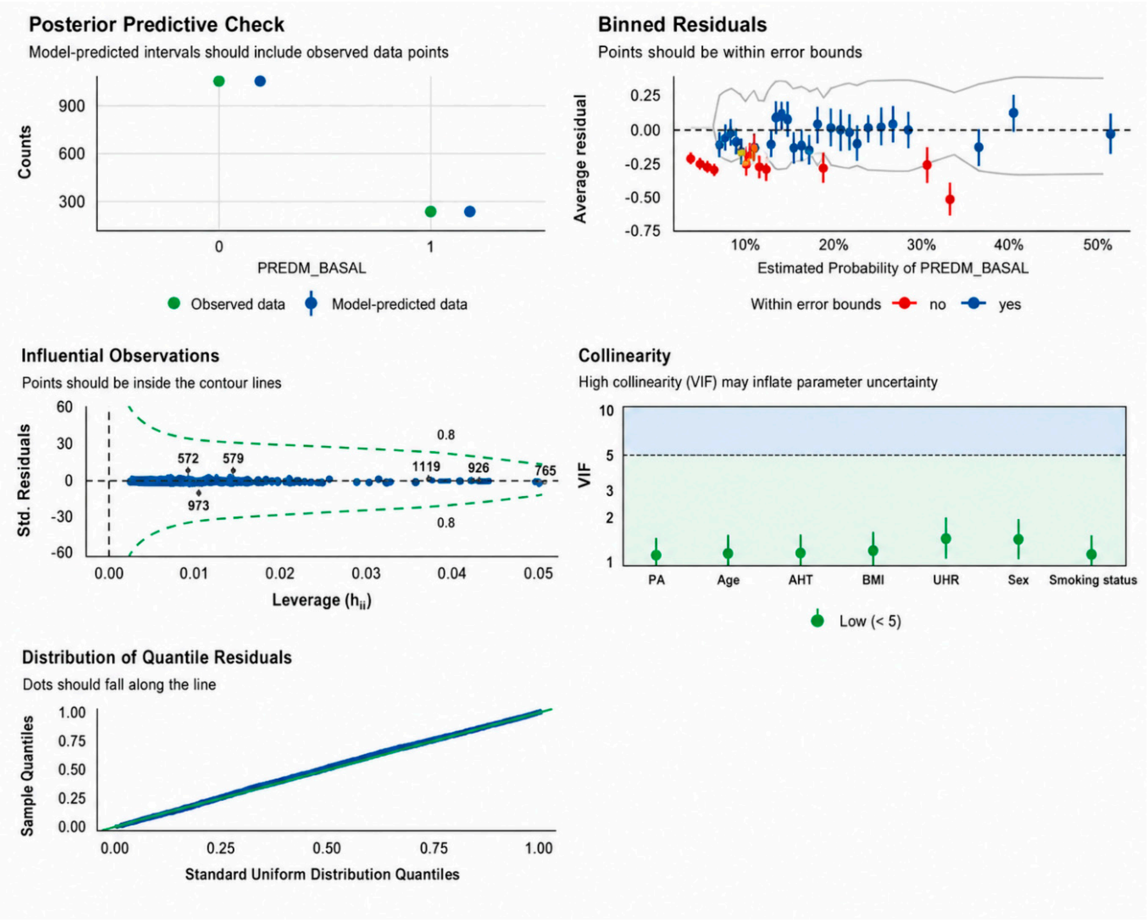

Abbreviations: PA, physical activity; CT-TAT, non-contrasted computed tomography total adipose tissue; AHT, arterial hypertension; BMI, body mass index; UHR, Uric acid to high-density lipoprotein cholesterol ratio.

Supplementary Table S2. DeLong test comparisons between UHR and other metabolic indices for the identification of prediabetes.

Group	Comparison	AUROC UHR	AUROC Comparator	Difference	p-value
Women	UHR vs HOMA-IR	0.713	0.804	-0.091	0.003
Women	UHR vs Insulin	0.713	0.710	0.003	0.920
Women	UHR vs Adipo-IR	0.713	0.704	0.009	0.792
Women	UHR vs HDL-C	0.713	0.641	0.072	<0.001
Women	UHR vs Uric acid	0.713	0.687	0.025	0.275
Men	UHR vs HOMA-IR	0.541	0.797	-0.256	<0.001
Men	UHR vs Insulin	0.541	0.711	-0.169	<0.001
Men	UHR vs Adipo-IR	0.541	0.710	-0.169	<0.001
Men	UHR vs HDL-C	0.541	0.562	-0.021	0.281
Men	UHR vs Uric acid	0.541	0.522	0.020	0.326
Total	UHR vs HOMA-IR	0.624	0.800	-0.175	<0.001
Total	UHR vs Insulin	0.624	0.709	-0.085	<0.001
Total	UHR vs Adipo-IR	0.624	0.697	-0.072	0.005
Total	UHR vs HDL-C	0.624	0.606	0.018	0.144
Total	UHR vs Uric acid	0.624	0.603	0.022	0.112

DeLong tests were performed to compare correlated ROC curves. Positive differences indicate higher AUROC values for UHR relative to the comparator marker, whereas negative differences indicate lower AUROC values for UHR. UHR, uric acid-to-HDL cholesterol ratio; HDL-C, high-density lipoprotein cholesterol; Adipo-IR, adipose tissue insulin resistance index; HOMA-IR, homeostatic model assessment of insulin resistance.
